# Unfavourable expression of pharmacologic markers in mucinous colorectal cancer

**DOI:** 10.1038/sj.bjc.6602330

**Published:** 2005-01-18

**Authors:** S C Glasgow, J Yu, L P Carvalho, W D Shannon, J W Fleshman, H L McLeod

**Affiliations:** 1 Department of Surgery, Washington University School of Medicine, St Louis, MI, USA; 2 Department of Medicine, Washington University School of Medicine, St Louis, MI, USA; 3 Department of Biostatistics, Washington University School of Medicine, St Louis, MI USA; 4 The Siteman Cancer Center, St Louis, MI, USA; 5 Department of Genetics, Washington University School of Medicine, St Louis, MI USA

**Keywords:** mucinous adenocarcinoma, colorectal neoplasms, pharmacogenomics, messenger ribonucleic acid, 5-fluorouracil, irinotecan, oxaliplatin, adjuvant chemotherapy

## Abstract

Patients with mucinous colorectal cancer generally have worse prognoses than those with the nonmucinous variety. The reason for this disparity is unclear, but may result from a differential response to adjuvant chemotherapy. We examined known molecular markers for response to common chemotherapy in these two histological subtypes. In all, 21 patients with mucinous and 30 with nonmucinous Dukes C colorectal cancer were reviewed for demographic data and outcome. Total RNA from the tumours and adjacent normal mucosa was isolated and reverse transcribed. Quantitative expression levels of drug pathway genes were determined using *TaqMan* RT–PCR (5-fluorouracil (5-FU): *TYMS*, *DPYD*, *ECGF1*; oxaliplatin: *GSTP1* (glutathione *S*-transferase pi), *ERCC1* and *2*; irinotecan: *ABCB1*, *ABCG2*, *CYP3A4*, *UGT1A1*, *CES2*, *TOP1*). Mucinous tumours significantly overexpressed both TYMS and GSTP1 relative to nonmucinous tumours and patient-matched normal mucosa. No significant differences in expression of the remaining markers were found. Mean follow-up was 20 months; 17 patients had recurrent disease. Among patients receiving 5-FU, those with mucinous tumours experienced shorter disease-free survival (DFS) than those with nonmucinous tumours (median DFS 13.8 *vs* 46.5 months, *P*=0.053). Mucinous colorectal cancer overexpresses markers of resistance to 5-FU and oxaliplatin. Likewise, DFS may be decreased in patients with mucinous tumours who receive 5-FU. The presence of mucin should be carefully evaluated in developmental trials of new agents for treating colorectal cancer.

Most evidence suggests that colorectal adenocarcinomas that produce an abundance of the glycoprotein mucin portend a worse clinical prognosis (Umpleby *et al*, 1985; Green *et al*, 1993; Nozoe *et al*, 2000), although this finding has not been consistently established (Sasaki *et al*, 1987; Halvorsen and Seim, 1988). Comprising between 10 and 15% of all colorectal adenocarcinomas, these tumours have a mucin content of at least 60% of tumour volume, typically at the advancing edge. The reasons for the worse prognosis with mucinous tumours are not clear, but possibly include difficulty obtaining complete resections (Umpleby *et al*, 1985), propensity for early spread to regional lymph nodes (Nozoe *et al*, 2000), and detection at more advanced stages of disease (Halvorsen and Seim, 1988). There also may be a higher proportion of mucinous tumours in familial cancer syndromes; approximately 30% of mucinous tumours demonstrate microsatellite instability, a defining trait of hereditary nonpolyposis colorectal cancer (Kakar *et al*, 2004). The responsiveness to adjuvant chemotherapy of mucinous adenocarcinoma specifically has not been examined.

Various genes are known to influence tumour response or patient survival following chemotherapy (McLeod and Murray, 1999). Their products consist of intracellular drug targets, cell-membrane transporters, components that detoxify or metabolise, and DNA repair enzymes. *TYMS* encodes thymidylate synthase, a key enzyme in pyrimidine metabolism. Numerous studies have demonstrated that overexpression of *TYMS* correlates with poor response to 5-fluorouracil (5-FU) (Johnston *et al*, 1995; Leichman *et al*, 1997; Salonga *et al*, 2000; Iacopetta *et al*, 2001; Kornmann *et al*, 2002). Likewise, elevated levels of other enzymes in the 5-FU pathway (namely, *DPYD* (dihydropyrimidine dehydrogenase) and *ECGF1* (thymidine phosphorylase)) have been shown to affect epithelial tumour sensitivity (Etienne *et al*, 1995; Nita *et al*, 1998), clinical response (Metzger *et al*, 1998a), and time to recurrence (Salonga *et al*, 2000) following treatment with 5-FU. Additionally, expression levels of *DPYD* and *ECGF1* appear to be coregulated, particularly in metastatic colorectal cancer (Collie-Duguid *et al*, 2001).

Platinum agents such as oxaliplatin rely on the inability of nucleotide excision repair genes within the tumour to successfully remove bulky DNA adducts. Polymorphisms in *ERCC1* (excision repair crosscomplementing isoform 1) have been related to decreased survival in patients with colorectal cancer (Park *et al*, 2001). Similarly, expression of *ERCC2* (excision repair crosscomplementing isoform 2) in gastric cancers predicts response and survival (Metzger *et al*, 1998b). *GSTP1* (glutathione *S*-transferase pi) is a major route of detoxification of platinum agents. It is highly overexpressed in colon cancer, and drug-resistant tumours have elevated levels (Stoehlmacher *et al*, 2002).

Irinotecan is bioactivated to a more potent form via *CES2* (carboxylesterase 2), and colorectal adenocarcinomas overexpressing *CES2* are more sensitive to the drug (Wu *et al*, 2002). Irinotecan causes double-strand DNA breaks by binding to topoisomerase-I (*TOP1*) (Xu and Villalona-Calero, 2002), making it a key intracellular target. Members of the ATP-binding cassette superfamily of proteins (*ABCB1* (Patel and Mitra, 2001; Gottesman *et al*, 2002) and *ABCG2* (Wierdl *et al*, 2003)) function to export irinotecan from tumour cells, while drug metabolism via the cytochrome *P*450 system (*CYP3A4* (Kehrer *et al*, 2002; Xu and Villalona-Calero, 2002)) or glucuronidation (*UGT1A1* (Iyer *et al*, 1999, 2002)) can also occur.

In the current study, we examined expression of the above 12 genes in colorectal cancer in order to characterise possible differences in response between the mucinous and nonmucinous subtypes of colorectal cancer. These genes were selected as likely candidates affecting clinical response to common adjuvant chemotherapy (i.e. 5-FU, irinotecan, and oxaliplatin) administered following resection of locally advanced tumours. A secondary goal was to correlate gene expression with disease-free survival (DFS), in the context of histological subtype.

## MATERIALS AND METHODS

In all, 51 Dukes stage C colorectal cancer specimens were selected from the Tissue Procurement Core of the Siteman Cancer Center, Washington University, St Louis, MO, USA for genetic evaluation. No tumours from patients with known inflammatory bowel disease or familial cancer syndromes (familial adenomatous polyposis or hereditary nonpolyposis colon cancer) were included. In total, 21 mucinous adenocarcinomas were selected, histologically defined by a mucin content exceeding 60% of the total tumour volume. The remaining 30 nonmucinous tumours were randomly chosen from a contemporary selection of 145 available Dukes' C colorectal cancer specimens. Adjacent normal mucosa was available for all tumours for comparison with the malignant epithelial component. Tissue was snap-frozen in liquid nitrogen immediately following surgical resection and stored at −80°C. Macrodissection was performed on all tumour specimens to ensure high tumour cellularity (median 86.3%, 65–95%). Clinical data were extracted from patients' medical records and clinic charts. The median clinical follow-up was 17.2 months (mean 20, range 2–59 months). Six patients with rectal cancer received neoadjuvant external beam radiation (total dose range 2000–4500 cGy). Patients gave informed consent for tumour banking, collection of clinical information, and subsequent decoded genetic analysis, as approved by the Washington University Human Studies Committee and the Siteman Cancer Center Protocol Review and Monitoring Committee.

Total RNA was extracted from the specimens using the Stratagene MicroRNA Isolation kit (Stratagene, La Jolla, CA, USA), and RNA was quantified with spectrophotometry. Reverse transcription to cDNA was performed using StrataScript reverse transcriptase with RNase Block Ribonuclease Inhibitor (Stratagene) and Oligo(dT) primers. *TaqMan* real-time PCR was performed on the ABI Prism 7900 Sequence Detector System (ABI, Foster City, CA, USA) with gene-specific dual-fluorescence-labelled oligonucleotide probes (Table 1). Each reaction well contained 10 ng cDNA, JumpStart *Taq* ReadyMix 2 × (5 *μ*l, Sigma-Aldrich, St Louis, MO, USA), forward and reverse primers 10 *μ*M (0.5 *μ*l each), fluorescence-labelled oligonucleotide 5 *μ*M (0.4 *μ*l), ROX passive reference dye 100 × (0.1 *μ*l), and molecular-grade water for a total reaction volume of 10 *μ*l. RT–PCR was performed with the following parameters: 50°C for 2 min to activate UNG enzyme, 95°C for 10 min to denature UNG and activate DNA polymerase, 40 cycles at 95°C for 20 s and 60°C for 1 min. All specimen reactions were performed in triplicate with appropriate nontemplate controls, and the coefficient of variation (CV) was less than 5% for all replicates.

Relative gene expression was calculated using a modified comparative threshold cycle (*C*_T_) method, as described previously (Pfaffl, 2001; Yu *et al*, 2003). This method accounts for varying RT–PCR amplification efficiencies between different primer/probe sets, rather than assuming all genes of interest amplify at an equal rate. Amplification efficiency (*E*) was determined from standard dilution curves for each primer/probe set. *β*-actin was used as the internal reference gene, as it had no significant variation between tumour and normal samples, and less than three-fold variation among all samples. Expression is given as a unit-less measure relative to the calibrator specimen that had the highest *C*_T_ (i.e. the 1 × sample), using the following equation: 
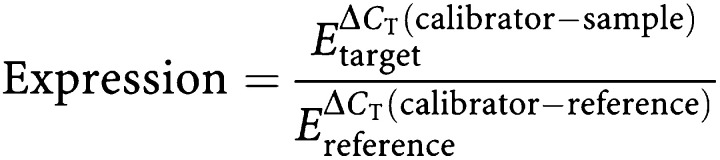


All statistical analyses were performed with GraphPad InStat (GraphPad Software, San Diego, CA, USA). The Mann–Whitney and Fisher's exact tests were utilised as appropriate. Comparisons between tumour samples and patient-matched normal mucosa were made using the Wilcoxon test for matched pairs. Survival curves were generated using the Kaplan–Meier method, with significance assessed by the log-rank test. A *P*-value less than 0.05 was considered significant. Values are expressed as mean [−95% confidence interval (CI), +95% CI].

## RESULTS

Patient characteristics are depicted in Table 2. All patients underwent resection with curative intent. As previously mentioned, all patients had positive lymph node metastases (AJCC stage III). There were no significant differences between the mucinous and nonmucinous groups for age at diagnosis, gender, tumour size, histologic grade, or number of positive lymph nodes. No mucinous tumours occurred in the rectum, whereas 20% of the nonmucinous tumours were rectal. However, differences in tumour location did not reach statistical significance. A total of 13 mucinous tumours occurred proximal to the splenic flexure, compared to 14 nonmucinous tumours (*P*=0.58).

**Figure 1:**
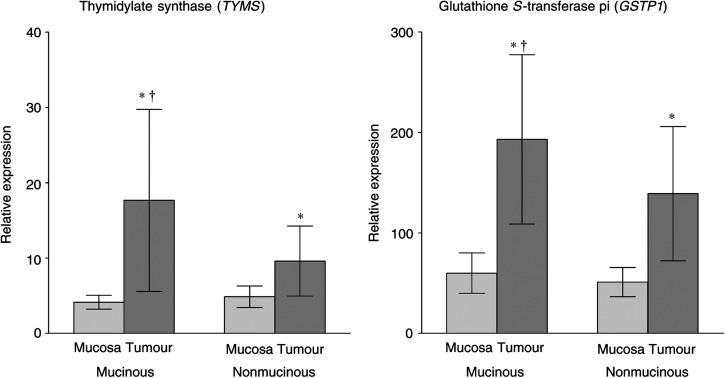
Differential expression of *TYMS* and *GSTP1* in mucinous and nonmucinous colorectal adenocarcinoma. ^*^P<0.001 relative to patient-matched normal mucosa; ^†^*P*<0.05 relative to nonmucinous adenocarcinoma. Error bars represent the 95% CI of the mean.

Relative gene expression of *DPYD*, *ECGF1*, *ERCC1*, *ERCC2*, *ABCB1*, *ABCG2*, *CYP3A4*, *UGT1A1*, *CES2*, and *TOP1* did not differ significantly between mucinous and nonmucinous tumours, although the difference in *ERCC2* expression approached statistical significance (*P*=0.083), with higher expression in the mucinous subgroup. However, as depicted in Figure 1, *TYMS* expression was significantly higher in mucinous tumours compared to nonmucinous tumours (17.7 arbitrary units [95% CI 5.8, 30.0] *vs* 9.6 [5.0, 14.3] respectively, *P*=0.013). Overexpression of *TYMS* was also observed in both subtypes of adenocarcinoma compared to normal mucosa. Relative to patient-matched mucosa, nonmucinous tumours demonstrated less overexpression (tumour mean 9.6 [5.0, 14.3] *vs* mucosal mean 4.9 [3.5, 6.3], *P*=0.01) than the mucinous variant (tumour mean 17.7 [5.8, 30.0] *vs* mucosal mean 4.1 [3.2, 5.1], *P*<0.001). There was no difference in nonmalignant mucosal expression of *TYMS* between patients with mucinous and nonmucinous cancers.

*GSTP1* expression was also significantly greater in mucinous tumours (193.0 [108.8, 277.3]) than in nonmucinous tumours (139.0 [72.2, 205.8], *P*=0.029). Both histologic tumour subtypes had higher expression of *GSTP1* than the matched normal mucosa. The degree of overexpression in mucinous tumours (tumour mean 193.0 [108.8, 277.3] *vs* mucosal mean 60.0 [39.9, 80.0], *P*<0.001) was greater than in nonmucinous tumours (tumour mean 139.0 [72.2, 205.8] *vs* mucosal mean 51.0 [36.4, 65.6], *P*<0.001). There was no difference in nonmalignant mucosal expression of *GSTP1* in patients with mucinous and nonmucinous cancers.

**Figure 2:**
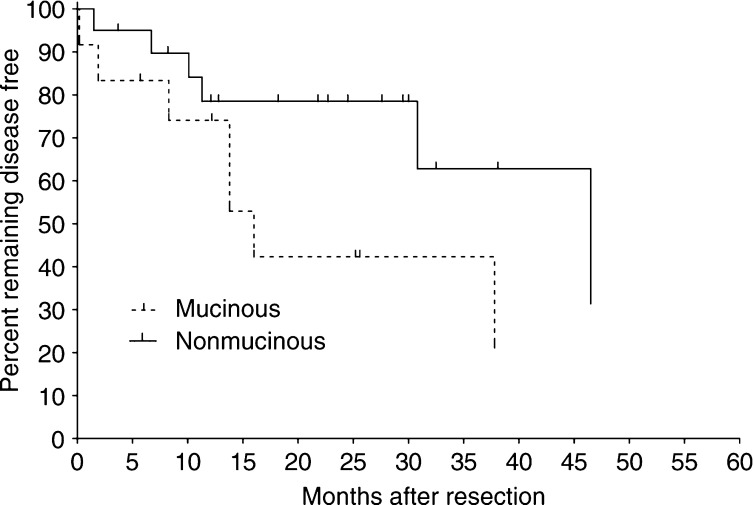
Disease-free survival based on histologic subtype for all patients who received adjuvant 5-FU (*N*=30 total; *P*=0.053).

Clinical follow-up data were available for 44 of the 51 patients (86%); those without complete data were excluded from further analyses. Of the 44 patients, 17 experienced recurrence. Time to disease recurrence was evaluated for mucinous and nonmucinous tumours. Disease-free survival between the two groups was not significantly different, with median times to recurrence of 54.2 and 46.5 months (*P*=0.5) for patients with mucinous and nonmucinous adenocarcinoma, respectively. In all, 14 patients either did not complete a course of primary 5-FU-based chemotherapy (*N*=8) or were treated with other first-line treatment (*N*=6, irinotecan or neoadjuvant radiation only for rectal cancer). The median DFS for all 14 patients did not differ significantly from the entire cohort (52 months), nor was there a detectable difference between those with mucinous or nonmucinous tumours. However, subset analysis of the 30 patients who received 5-FU as primary adjuvant treatment revealed a shorter time to recurrence in patients with mucinous tumours (Figure 2). The median DFS was 13.8 months in the population of patients with mucinous adenocarcinoma (*N*=11) who received 5-FU chemotherapy. Conversely, patients with nonmucinous tumours (*N*=19) who underwent 5-FU treatment had a median DFS of 46.5 months; the trend of shorter time to recurrence in these patients approached statistical significance (*P*=0.053). Among patients treated with 5-FU, those with mucinous tumours had significantly higher *TYMS* expression (mean 20.8 *vs* 7.4 for patients with nonmucinous tumours who received 5-FU, *P*=0.014). Owing to limited patient numbers and clinical follow-up, subset analysis of patients receiving other chemotherapeutic agents (i.e. oxaliplatin or irinotecan) could not be performed. There were no differences in recurrence or survival when patients who were treated with adjuvant chemotherapy were stratified based on whether their tumour expressed greater than or less than the median level of either *TYMS* or *GSTP1*.

## DISCUSSION

It is generally recognised that mucinous adenocarcinomas of the colon and rectum have a worse prognosis following resection than nonmucinous tumours (Symonds and Vickery, 1976; Umpleby *et al*, 1985; Green *et al*, 1993; Consorti *et al*, 2000; Nozoe *et al*, 2000). Compared to the more common nonmucinous variety, mucinous tumours metastasise to lymph nodes with increased frequency (Nozoe *et al*, 2000), are more prone to local recurrence (Umpleby *et al*, 1985; Connelly *et al*, 1991) and peritoneal carcinomatosis (Nozoe *et al*, 2000), are less likely to be resected with negative margins (Umpleby *et al*, 1985), and are typically diagnosed at an advanced stage (Younes *et al*, 1993). They also have altered p53 expression (Hanski *et al*, 1996) and increased microsatellite instability (Messerini *et al*, 1997). Although mucinous tumours occur more frequently in the proximal colon, left-sided and rectal mucinous tumours have an overall worse prognosis relative to location-matched nonmucinous tumours (Green *et al*, 1993). This finding may be at least partially explained by over-expression of both p53 (Lenz *et al*, 1996) and thymidylate synthase (Allegra *et al*, 2002) in left-sided tumours, although no previous study has examined expression in the mucinous subset alone. Based on these genetic differences and its more aggressive clinical behaviour, some authors have suggested that mucinous adenocarcinomas should be categorised and treated as a biological entity distinct from other colorectal adenocarcinomas (Consorti *et al*, 2000). The current study confirms the biological difference between mucinous and nonmucinous tumours. We found significantly higher expression of thymidylate synthase (*TYMS*) and *GSTP1* in mucinous colorectal adenocarcinomas than in equivalent-stage nonmucinous tumours. Both mucinous and nonmucinous tumours also overexpressed these genes relative to normal colonic mucosa.

The association between high levels of *TYMS* in tumours and poor clinical response following 5-FU treatment of colorectal cancer is well documented (Johnston *et al*, 1995; Leichman *et al*, 1997; Salonga *et al*, 2000; Iacopetta *et al*, 2001). Elevated expression has also been correlated with time to recurrence in patients with stage II or III disease (Kornmann *et al*, 2002). The finding of overexpression of *TYMS* in mucinous tumours in our study offers a possible explanation as to why patients with these tumours tend to do worse. To our knowledge, response of mucinous tumours to 5-FU treatment has not been specifically investigated. In the recent literature regarding mucinous adenocarcinoma, the large majority of patients with locally advanced colorectal cancer would have received 5-FU. Perhaps decreased response to 5-FU due to overexpression of *TYMS* contributes to the relatively poorer prognosis for patients with mucinous adenocarcinoma. Among patients in our study who received 5-FU following initial surgical resection, there was a trend towards increased DFS only in the nonmucinous tumour group. Although the *P*-value did not reach the significance threshold of 0.05, this finding intuitively makes sense, given the fact that tumours in the nonmucinous group had significantly lower expression of *TYMS*. Owing to the small number of patients who either did not complete 5-FU-based chemotherapy or received other adjuvant treatment, no definitive conclusions can be drawn.

Oxaliplatin causes cell death by forming bulky, DNA helix-distorting adducts via crosslinking between guanine bases. *GSTP1* belongs to the glutathione *S*-transferase superfamily and is a major mechanism of detoxification of platinum agents. It is highly overexpressed in colorectal cancer, with increased levels in drug-resistant tumours (Stoehlmacher *et al*, 2002). In the current study, *GSTP1* expression was found to be similarly elevated in malignant tissue relative to normal colonic mucosa. Interestingly, expression was significantly greater in mucinous tumours. As our cohort of tumours was assembled in a retrospective manner from historically collected samples, none had been treated with oxaliplatin. However, the finding of elevated *GSTP1* expression within mucin-producing tumours suggests that diminished clinical response may be expected from oxaliplatin-treated tumours. Further research examining this hypothesis is warranted.

Regarding the remaining ten markers, there were no significant differences in gene expression between mucinous and nonmucinous colorectal cancers. This negative finding does not imply they do not have a role in determining outcome following chemotherapy. Rather, they may not contribute to the differing clinical responses seen in the treatment of mucinous adenocarcinoma relative to the nonmucinous variant. In fact, it would be expected that altered expression of some genes contributes to the general drug-resistant phenotype seen in all colorectal adenocarcinomas, while expression of others distinguishes individual biologically distinct subtypes and determines their relative clinical responsiveness.

The intent of the current study was to examine the relative expression in colorectal cancer of certain genes believed to influence clinical response to adjuvant chemotherapy. It was not designed to characterise extensively the relationships between gene expression, response to chemotherapy, and patient outcome. The clinical follow-up data were limited, with a median follow-up of only 17.2 months. Since this is a retrospective study, chemotherapy selection and dosing were not standardised. Additionally, rectal cancers were somewhat under-represented, comprising 20% of the nonmucinous cohort *vs* approximately 30% of all colorectal adenocarcinomas. Nonetheless, several significant associations were found that may lead to further investigations. Longer patient follow-up and standardised treatment will be required to fully explore the role of *TYMS* and *GSTP1* overexpression in mucinous adenocarcinoma. Protein analysis of these enzymes may validate our findings and provide further insight into their complex intracellular interactions. However, much of the data supporting the negative influence of overexpression of both *TYMS* and *GSTP1* on patient outcome has been generated by examining either germline polymorphisms or transcriptional levels (Metzger *et al*, 1998a; Salonga *et al*, 2000; Stoehlmacher *et al*, 2002).

In addition to pharmacogenetic markers for outcome following specific drug treatment, there are a variety of patient characteristics (e.g. lymph node metastases, age), histologic findings (e.g. neurovascular invasion, differentiation), and genetic factors (e.g. p53 expression, microsatellite instability), which appear to influence patient prognosis in colorectal cancer. We chose not to perform an exhaustive examination of all such prognostic factors, but instead to examine the heterogeneity and differential expression of known pharmacogenetic markers in mucinous adenocarcinomas. Whether other markers of prognosis such as microsatellite instability can be utilised clinically to predict outcome in mucinous colorectal cancer remains to be determined.

In conclusion, mucinous tumours significantly overexpressed *TYMS* and *GSTP1* relative to both normal mucosa and to nonmucinous adenocarcinomas. Both these genes are known markers for resistance to chemotherapy. Among those who received adjuvant 5-FU, DFS was shorter in patients with mucinous tumours. It is unclear whether the overexpression of *TYMS* contributed to these earlier recurrences. Clinical response may be improved for mucinous colorectal cancer if treated with combination chemotherapy or perhaps agents such as irinotecan, where no differences in drug pathway markers were identified. The mucin status of colorectal tumours should be carefully addressed in future trials of novel treatments.

## Figures and Tables

**Table 1:** *TaqMan* real-time PCR primers and probes

***Gene* (NCBI LocusLink ID)**	**Sequence**	**Reporter dye**
*ABCB1* (5243)	GCTGGCACAGAAAGGCATCT	
	CAGAGTTCACTGGCGCTTTG	
	TCCAGCCTGGACACTGACCATTGAAA	TET
*ABCG2* (9429)	CAGGTCTGTTGGTCAATCTCACA	
	CATATCGTGGAATGCTGAAGTACTG	
	CCATTGCATCTTGGCTGTCATGGC	TET
*CES2* (8824)	AATCCCAGCTATTGGGAAGGA	
	CTGGCTGGTCGGTCTCAAAC	
	TGGCCTCAAGCCATCCTCCCATCT	TET
*CYP3A4* (1576)	TCTCCTTTCATATTTCTGGGAGACA	
	GCATCGAGACAGTTGGGTGTT	
	TGTTTCCCTACACCTCTTGCATTCCATCCT	TET
*DPYD* (1806)	ATCTTGAATCGTTGGATCTAGATCGA	
	GAGTAGTAGCTAGTCGCTACTGAT	
	ACCGTACTCATCGTAGCTACCATGAA	TET
*ECGF1* (1890)	GGTTCCTGCGGACGGAAT	
	GAGTAGTAGCTAGTCGCTACTGAT	
	CAGCCAGAGATGTGACAGCCACCG	TET
*ERCC1* (2067)	TACCCCTCGACGAGGATGAG	
	CAGTGGGAAGGCTCTGTGTAGA	
	CCTGGAGTGGCCAAGCCCTTATTCC	TET
*ERCC2* (2068)	TTGGCGTCCCCTACGTCTAC	
	CTGGTCCCGCAGGTATTCC	
	CACAGAGCCGCATTCTCAAGGCG	FAM
*GSTP1* (2950)	CCTCACCCTGTACCAGTCCAATA	
	TCCTGCTGGTCCTTCCCATA	
	TCACCTTGGGCCGCACCCTTG	TET
*TOP1* (7150)	GGCGAGTGAATCTAAGGATAATGAA	
	TGGATATCTTAAAGGGTACAGCGAA	
	ACCATTTTCCCATCATCCTTTGTTCTGAGC	TET
*TYMS* (7298)	GCCTCGGTGTGCCTTTCA	
	CGTGATGTGCGCAATCATG	
	CATCGCCAGCTACGCCCTGCTC	TET
*UGT1A1* (7361)	TTGGGAGTGCGGGATTCA	
	AGATAAGATTAAAACTGCCATTTGCA	
	TGGTCCCACCGCTGCCCCTA	TET

Sequences are listed as forward, reverse, and probe oligonucleotides, in the 5′ to 3′ direction.

**Table 2:** Patient characteristics

	**Mucinous**	**Nonmucinous**	***P*-value**
*N*	21	30	—
Age (years)	68.9	69.2	0.98
Male:female	12:9	16:14	1.00
Tumour diameter (cm)	5.3	5.1	0.31
			
*Location*			0.16
Right	9 (43%)	12 (40%)	—
Transverse	4 (19%)	3 (10%)	—
Left/sigmoid	8 (38%)	9 (30%)	—
Rectum	0 (0%)	6 (20%)	—
			
Median no. LNs evaluated	19	16.5	0.52
Median no. positive LNs	2	2	0.78
			
*Tumour differentiation*			0.71
Poor	5 (24%)	7 (23%)	—
Moderate	13 (62%)	16 (53%)	—
Well	3 (14%)	7 (23%)	—

All patients are stage III.
